# Shredded-Coconut-Derived Sulfur-Doped Hard Carbon via Hydrothermal Processing for High-Performance Sodium Ion Anodes

**DOI:** 10.3390/nano15100734

**Published:** 2025-05-14

**Authors:** Yuanfeng Liu, Shuai Chen, Chengzhi Zhang, Guochun Li, Junfeng Liu, Yong Wang

**Affiliations:** Institute for Energy Research, Jiangsu University, Zhenjiang 212013, China; 2222212080@stmail.ujs.edu.cn (Y.L.); 2222393015@stmail.ujs.edu.cn (S.C.); 2222393023@stmail.ujs.edu.cn (C.Z.); liguochun@ujs.edu.cn (G.L.)

**Keywords:** biomass, hard carbon, sodium-ion battery, anode, hydrothermal, sulfur, doping

## Abstract

The extensive use of sodium-ion batteries has made it important to develop high-performance anode materials. Owing to their good sustainability, low cost, and excellent electrochemical properties, hard carbon materials are expected to be a good choice, especially biomass-derived hard carbon. In this study, we successfully synthesized a coir-based carbon nanosphere as an anode material. The hard carbon has a low degree of structural ordering, small particle size, and multiple pore networks for easy sulfur doping compared to the conventional direct high-temperature sulfur doping. The material has a high reversible capacity of 536 mAh g^−1^ and an initial Coulombic efficiency of 53%, maintaining a reversible capacity of 308 mAh g^−1^ at a high current density of 5 A g^−1^, achieving a capacity retention of 90.3% after 1000 cycles. The performance enhancement stems from a combination of enlarged layer spacing, an increased specific surface area, enhanced porosity, and doped sulfur atoms. This study provides an effective strategy for the conversion of biomass waste into high-performance sodium-ion anode material batteries.

## 1. Introduction

In recent years, the extensive use of fossil energy has caused environmental pollution, and the demand for renewable energy has gradually increased. Lithium-ion batteries have become the preferred choice for storing renewable energy due to their great energy density and good cycle time, and they have captured a large market [1]. However, the application of lithium batteries is hampered by the lack of lithium resources and the fact that its distribution is mainly in South America. In contrast, sodium-ion batteries (SIBs) have received widespread attention due to their high content and wide distribution [2,3,4,5]. Sodium-ion batteries are constrained by their specific capacity and cycling capability, and thus, solving these problems is of benefit for the efficient use of resources [6].

Choosing suitable sodium-ion anode materials can improve sodium-ion battery performance. Lithium ions have a diameter of 0.76 Å, making graphite, with its stable structure, an excellent anode material. However, sodium ions have a diameter of 1.02 Å, making it difficult to embed them into the conventional graphite layer, leading to a decrease in their specific capacity and limiting their commercial use. Therefore, choosing the proper anode material becomes even more important. There have been extensive studies on biomass materials in various fields, such as sorbent [7,8], biofuel [9,10], and biomedical applications [11]. With the advancement of new energy technologies, biomass materials are also increasingly being applied in the field of energy materials. Biomass material is expected to be a suitable anode material due to its low price, environmental friendliness, and rich pore structure and wide layer [12,13,14], spacing conducive to the storage of sodium ions. Many biomass materials, including agricultural residues, basswood [15], coffee grounds [16], switchgrass [17], lotus roots [18], and Prunus armeniaca seeds [19], are effective anode materials for sodium ions in research studies. However, their specific capacity is still relatively low.

Heteroatom doping, such as nitrogen [20,21,22,23,24], oxygen [25], phosphorus [26,27,28], fluorine [29], boron [27,30], and sulfur [31,32,33,34], significantly improves the ability of biomass materials to store sodium ions. Among these atoms, sulfur atoms can provide an additional Ferrari effect and increase the layer spacing, which can more significantly improve the energy density and multiplicity performance of biomass materials [35]. The doping of sulfur atoms increased the interlayer spacing of garlic, in addition to forming a rich pore structure. The active sites were enhanced, possessing a specific capacity of 605 mAh g^−1^ at 0.05 A g^−1^ [33]. The sulfur-doped high-temperature carbonization of biomass materials produces a porous structure, leading to an increase in the initial coulombic efficiency (ICE) and a significant improvement in the multiplicity performance. At the same time, nitrogen–sulfur co-doping plays a synergistic role, making it more effective in increasing the conductivity and porosity of carbon materials, improving the electrochemical performance [32]. After theoretical calculations, the doping of sulfur atoms accelerates the reaction kinetics and increases the transport rate of sodium ions [31].

Hydrothermal treatment can effectively increase the porosity of carbon materials, improving the porosity of carbon materials [36,37]. The hydrothermal treatment of the wood fibers removes inorganic impurities from the biomass, creating a more stable structure that significantly improves their multiplicative properties [38,39]. Switchgrass is hydrothermally enriched with a pore structure and suitable specific surface area, giving it a specific capacity of 313 mAh g^−1^ at a current density of 0.1 A g^−1^ and an ICE of 85% [17].

In this paper, we modified biomass carbon materials using hydrothermal pretreatment and sulfur doping through high-temperature heating, significantly improving the sodium-storage properties of coconut silk. Compared with the sulfur doping process without hydrothermal treatment, it has a richer pore structure and wider layer spacing. It was found that the synthesized biomass carbon material possessed a specific capacity of 536 mAh g^−1^ at a current density of 0.1 A g^−1^ and 230 mAh g^−1^ at a current density of 10 A g^−1^ when heated at a temperature of 500 °C. In addition, when the current density is 5 A g^−1^, even after 1000 cycles tests, it still has a 90% initial energy density, showing good cycling stability. This paper provides a method for converting biomass waste into high-performance sodium ion anode materials, increasing the economic value of biomass materials. It also offers a means to increase the sodium-storage capacity of sodium-ion batteries.

## 2. Experimental Sessions

### 2.1. Synthesis of Materials

The coconut shreds were cleaned using deionized water and ethanol and dried at 80 °C overnight. Then, the dried shredded coconut material was ball-milled for 4 h at 600 rpm. The ball-milled material was mixed well with 60 mL of a 6 M sulfuric acid solution (Sinopharm, Beijing, China) and heated in a PTFE-lined hydrothermal kettle (Tkareactor, Xian, China) for 2 h at a temperature of 180 °C. Subsequently, it was filtered and dried overnight. After mixing the dried carbon material with sulfur powder in ratio of 1:2 to give uniformity, it was placed in a tube furnace(carbolite-gero, Shanghai, China) and heated at 500, 700, and 900 °C heating temperatures for 3 h with a heating rate of 5 °C per min. Finally, the carbon materials were washed with deionized water and ethanol, yielding the final products named S-BC-HT-500, S-BC-HT-700, and S-BC-HT-900. The sulfur-doped samples synthesized at 500 °C without conducting hydrothermal treatment are recorded as S-BC-500, and the non-sulfur samples synthesized at 500 °C with hydrothermal treatment are recorded as BC-500.

### 2.2. Characterization of Materials

An X-ray (XRD, XRD 6100) (Shimadzu Corporation, Kyoto Prefecture, Japan) diffractometer was used to test its crystal structure and physical parameters with Cu *K_α_* radiation (λ = 1.5406 Å) in a 2θ range of 10–80°. Field emission electron microscopy (SEM, JEOL 7800F) (JEOL, Tokyo, Japan) and high-resolution transmission electron microscopy (TEM, Talos F200X G2) (Thermofisher, Waltham, MA, USA) were performed to analyze the surface morphology and microstructure of materials. The surface chemistry and electronic valence states were analyzed by using X-ray photoelectron spectroscopy (XPS, Thermo Scientific K-Alpha) (Thermofisher, Waltham, MA, USA). The specific surface area and pore structure were tested using a fully automated specific surface and porosity analyzer (Micromeritics ASAP 2460) (micromeritics, Atlanta, GE, USA) degassed at 200 °C for 6 h, using the nitrogen-desorption curve method. A thermogravimetric analysis (TG 209 F3 TARSUS) (NETZSCH, Selb, German) was performed to evaluate the stability of materials in a test environment under nitrogen gas.

### 2.3. Electrochemical Measurements

When making the electrode, the carbon material was mixed with conductive carbon black (super P) (Sinopharm, Beijing, China) and polyvinylidene fluoride (Sinopharm, Beijing, China) in a mass ratio of 8:1:1 [40]. It was dissolved in N-methyl-2-pyrrolidone (NMP) (Sinopharm, Beijing, China) and stirred for 4 h to obtain a homogeneous slurry. It was applied to the charcoal-coated copper foil using a four-sided preparator (Gonoava, Changsha, Hunan, China) and then dried overnight. It was made into electrodes using a roller press (szjkne, Shenzhen, China) with an electrode area of 1 cm^2^ and a load between 0.8 and 1.2 g cm^−2^. Coin cells (CR2032) were assembled in an argon-filled glove box (MBRAUN, Munich, German) (water-oxygen content always below 0.01 ppm). Sodium metal (Sinopharm, Beijing, China) was used as the cathode material, glass fibers (szjkne, Shenzhen, China) were used as the diaphragm, the electrolyte was 1 M NaClO_4_ (Sinopharm, Beijing, China), which was mixed with ethylene carbonate (EC) (Sinopharm, Beijing, China) and diethyl ethyl carbonate (DEC) (Sinopharm, Beijing, China) in a 1:1 ratio by volume, and 5 wt% of vinyl fluorocarbonate carbonate (FEC) (Sinopharm, Beijing, China)was used as the additive. Cyclic voltammetry (CV)and electrochemical impedance spectroscopy (EIS) were tested on the Ivium (Ivium, Eindhoven, Netherlands). Constant current charge/discharge tests and constant current intermittent titration tests (Gitt) were performed on a Neware CT-4008Tn (Neware, Shenzhen, China) with a discharge range from 0.01 to 3.0 V. Tests were conducted at room temperature (25 ± 2 °C).

## 3. Results and Discussion

**Figure 1:**
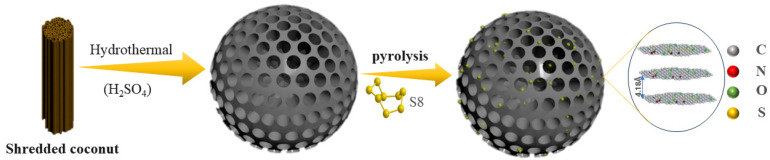
Schematic illustration of the synthesis process for sulfur-doped 3D carbon nanospheres.

As shown in Figure 1, the synthesis of sulfur-doped nanospheres consists of two main steps: first, the coconut silk is hydrothermally pretreated in a sulfuric acid solution, followed by pyrolysis with sulfur under an argon atmosphere at 500, 700, and 900 °C. The resulting materials are labeled S-BC-HT-500, S-BC-HT-700, and S-BC-HT-900, respectively.

**Figure 2:**
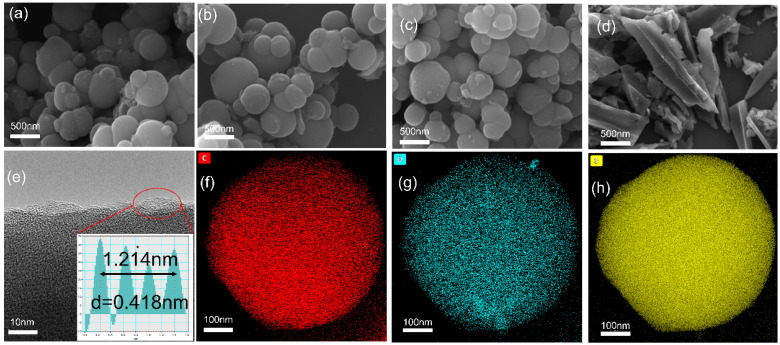
Microscopy characterizations of S-BC samples. (**a**–**d**) Scanning electron microscopy (SEM) images of S-BC-HT-500, 700, 900, and S-BC-500, respectively; (**e**) TEM images of S-BC-HT-500 sample showing its nanostructure and disordered carbon characteristics; (**f**–**h**) elemental mapping of S-BC-HT-500.

In the microscopic images, there are large differences between the samples. The sulfur-doped carbon material (S-BC-500) exhibits a bulk structure (Figure 2d), whereas the hydrothermally treated carbon material shows a regular spherical structure (Figure 2a–c). This difference is mainly due to hydrothermal treatment. Coconut silk is mainly composed of lignin, hemicellulose, and cellulose. During the hydrothermal process, the hydrolysis of shredded coconut occurs, in which the hydrolysis of hemicellulose and cellulose produces very small glucose molecules, which are rearranged and aggregated into relatively homogeneous spherical particles [41,42]. In addition, the acidic environment formed by sulfuric acid and the water-absorbing properties from sulfuric acid commonly accelerate the rate of hydrolysis of large molecules into small molecules in shredded coconut. Thus, the synthesized carbon materials mainly show a circular structure with particle diameters mainly in the nanometer range.

From the transmission electron microscopy (TEM) image of S-BC-HT-500 (Figure 2e), we can obtain the information that the material mainly exhibits an amorphous structure with microscopic pores. These microstructures, as well as the amorphous components, provide more active sites for sodium ion storage, increasing the sodium ion transport rate. The layer spacing is about 0.418 nm, which is significantly larger than the graphite (0.335 nm). Its layer spacing is more favorable for the insertion and insertion out of sodium ions, which reduces the spatial obstruction. Meanwhile, Energy-dispersive X-ray spectroscopy (EDS) images (Figure 2f–h) show that S, C, and O elements are uniformly distributed inside the carbon spheres, indicating that the sulfur doping is in the monodisperse doping and improves the overall stability.

**Figure 3:**
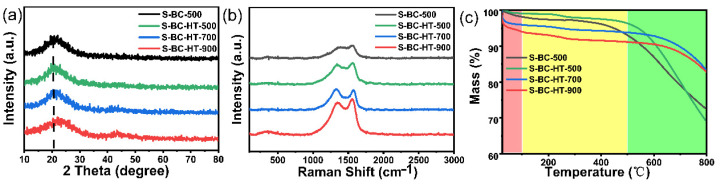
(**a**) XRD patterns; (**b**) Raman spectra; and (**c**) TGA of S-BC-HT-500, 700, 900, and S-BC-500 samples.

X-ray diffraction (XRD) was used to analyze the effect of hydrothermal and carbonization temperatures on the structure of the carbon layers (Figure 3a). The diffraction peaks mainly appear at 22.3° and 44.0°, corresponding to the (002) and (110) crystal planes of carbon. Their main manifestation is amorphous carbon, and the diffraction peak of S-BC-HT-500 showed a leftward shift, comparing it with S-BC-500, indicating a greater spacing of space structures after hydrothermal heating. As the temperature increases, their diffraction angle shifts to the right, which indicates a decrease in the layer spacing according to the Bragg equation (d = λ/2sinθ).

Disorder and structural defects in carbon materials can be tested via Raman spectroscopy, shown in Figure 3b. Two peaks appear in the spectrum: the D-band (1350 cm^−1^) associated with structural defects and the G-band (1570 cm^−1^) associated with graphite carbonization. *I_D_*/*I_G_* is an important parameter to respond to the disorder of carbon materials, which is calculated to be 0.89, 0.90, 1.01, and 0.85 for S-BC-HT-500, S-BC-HT-700, S-BC-HT-900, and S-BC-500. Comparing S-BC-HT-500 and S-BC-500, it can be seen that after hydrothermal treatment, it has a greater *I_D_*/*I_G_*, indicating a higher degree of disorder and more molecular pores. This may stem from the fact that hydrothermal disruption of the macromolecular structure produces a large number of defects and pores through the hydrolysis and aggregation of cellulose, which enhance the storage and transport capacity of sodium ions. Moreover, as the temperature increases, its *I_D_*/*I_G_* gradually increases, which is because when the temperature increases the volatilization of sulfur elements led to an increase in their defects. In addition, there are two peaks at 250 and 500 cm^−1^, which represent C–S, illustrating that the sulfur doping is mainly in the form of chemical bonding.

Thermogravimetric testing provides information on the thermal stability and sulfur content of the material (Figure 3c). The thermogravimetric curve can be divided into three regions: in 0–100 °C, the weight loss originates from water evaporation. From 200 to 500 °C, the weight of the samples changes a little, except for S-BC-500, which indicates that the material is not stable like the other materials, and the weight loss is a joint result of carbon and unbonded sulfur. Above 500 °C, all the samples start to lose weight: comparing S-BC-HT-500 and S-BC-500, the proportion of loss after hydrothermal treatment is greater and the rate of decrease is also greater, indicating a higher sulfur content, which is because hydrothermal treatment results in additional pore space and specific surface area. Therefore, the sulfur has a greater area to contact with the carbon, leading to a greater proportion of its doping. With the temperature increase, the rate and proportion decrease gradually. Because when the temperature increases, the C–S bond is gradually broken, and the sulfur is gradually volatilized, leading to a decreased proportion.

**Figure 4:**
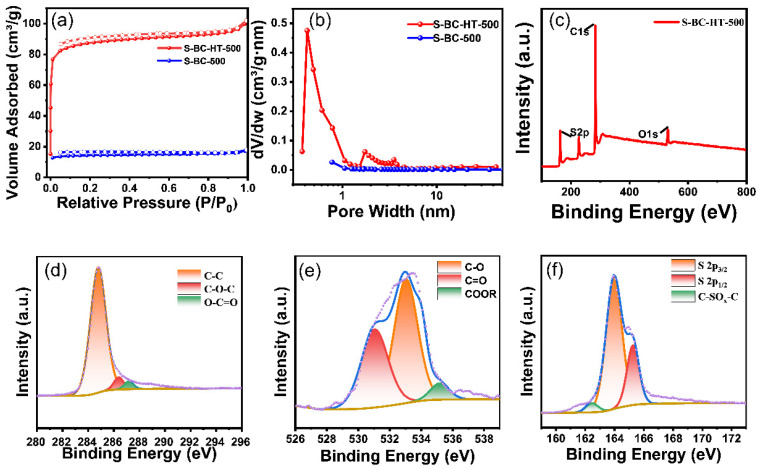
(**a**,**b**) The adsorption–desorption curves and pore size distributions of S-BC-HT-500 and S-BC-500 samples, respectively; (**c**) survey spectrum; (**d**) C 1s spectrum; (**e**) O 1s spectrum; and (**f**) S 2p spectrum.

The Brunauer-Emmett-Teller (BET) characterizations (Figure 4a,b) show that S-BC-HT-500 has abundant micropores and mesopores. Its specific surface area is 336 m^2^ g^−1^, which is significantly higher than the S-BC-500 of 54 m^2^ g^−1^, showing that hydrothermal treatment is favorable for the formation of pores. Its pore size distribution mainly exists between 0.3–3 nm (microporous) and 10–40 nm (mesoporous), and it forms a porous network structure. These hierarchical pore structures facilitate the diffusion of sodium ions and improve the interaction between the electrolyte and electrodes. The specific surface areas of BC-500, S-BC-HT-700, and S-BC-HT-900 were measured to be 326 m^2^ g^−1^, 356 m^2^ g^−1^, and 626 m^2^ g^−1^, respectively (Figure S1), suggesting that most of the sulfur is incorporated within the carbon network rather than located in the pores. The increase in the specific surface area with the rising temperature is due to the sulfur volatilization. The breakage of the carbon–sulfur bond leads to the formation of a defect where sulfur locates, which forms defects and pores in large quantities, thus increasing their specific surface area.

X-ray photoelectron spectroscopy (XPS) was used to analyze the elemental composition and valence distribution of S-BC-HT-500 (Figure 4c–f). As seen in these images, there are prominent peaks at 164, 284, and 532 eV, which correspond to the S 2p, C 1s, and O 1s orbitals. The C 1s spectrum was convolved into three peaks of 284.8, 286.4, and 287.2 eV They correspond to C–C, C–O, and C=O bonds, with the C–C bond dominating mostly between these bonds. Similarly, S 2p was convolved to 162.48, 163.98, and 165.28 eV, corresponding to the bonds C–SO_X_–C, S 2p3/2, and S 2p1/2. Finally, the peaks of O 1s correspond to 531.04, 533.03, and 534.05 eV, corresponding to the C=O, C–O, and COOR bonds.

**Figure 5:**
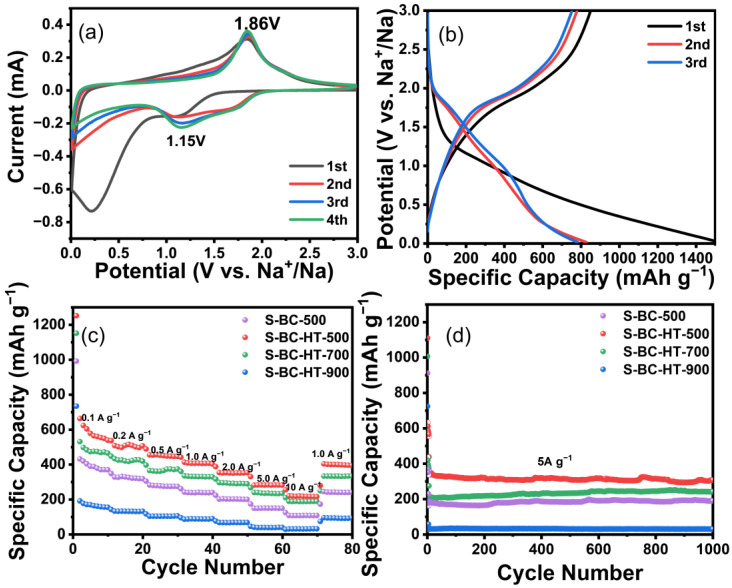
(**a**) The first four cycles of CV conducting at a scan rate V s^−1^ and (**b**) the first three cycles of GCD at 0.1A g^−1^ of the S-BC-HT-500 electrode. (**c**,**d**) The rate capacities in the range of 0.1-10 A g^−1^, as well as the long-term stability measurement at 5A g^−1^ of S-BC-HT-500, 700, 900, and S-BC-500 anodes, respectively.

By using electrochemical testing of S-BC-HT-500, S-BC-HT-700, S-BC-HT-900, and S-BC-500 to understand their electrochemical properties, cyclic voltammetry (CV) tests were performed on the S-BC-HT-500 at a sweep rate of 0.1 mV s^−1^. As shown in Figure 5a, an irreversible redox peak appeared in the interval around 0.25 V during the first cycle range, which disappeared in the second cycle, indicating that a solid electrolyte interface (SEI) forms in the first cycle. As the number of laps increases, the CV curves overlap well and gradually stabilize, showing the good stability of the anode materials. Respectively, the CV curves show different redox peaks at 1.86 V and 1.15 V, which is due to the redox reaction between sodium and sulfur. In the galvanostatic charge/discharge (GCD) curves (Figure 5b), there are discharge plateaus in the 1.5–2.2 V interval, which are consistent with the CV curves and show the contribution of the Faraday reaction to the capacity. 

Figure 5c shows the constant current charge/discharge tests at current densities of 0.1, 0.2, 0.5, 1, 2, and 5 A g^−1^ for different samples. It can be seen that the capacity of S-BC-HT-500 is 536, 497, 443, 403, 350, 280, and 216 mAh g^−1^, which is significantly higher than that of S-BC-500 (358, 317, 270, 235, 198, 146, and 105 mAh g^−1^) and BC-500 (87, 71, 50, 35, 23, 13, 9 mAh g^−1^) (Figure S2a). The capacity is still 216 mAh g^−1^ at a current density of 10 A g^−1^; meanwhile, when the current density is returned to 1 A g^−1^, its capacity still reaches 394 mAh g^−1^, indicating good rate performance and stability. Moreover, its ICE of 53% is higher than the S-BC-500′s 44.6% and BC-500′s 20.7%. The current density is set to 5 A g^−1^ (Figure 5d), and after 1000 cycles, the capacity of S-BC-HT-500 still reaches 308 mAh/g with a retention rate of 90.3%, which is higher than the 190 mAh g^−1^ value of S-BC-500 and the 13 mAh g^−1^ value of BC-500 (Figure S2b). This indicates that sulfur doping effectively enhances the initial efficiency and capacity. With the increase in temperature, its capacity decreases continuously, and at 900 °C, its capacity is only 153 mAh g^−1^ at a current density of 0.1 A g^−1^. The rapid decrease in the capacity is due to the decrease in sulfur content, showing that sulfur doping plays an important role in contributing to the capacity of carbon materials. Elemental analysis revealed that the sulfur contents of S-BC-HT-500, S-BC-HT-700, and S-BC-HT-900 samples are 31.32%, 24.48%, and 6.39%, respectively (Table S1), which is consistent with the observed trend in their electrochemical performance.

**Figure 6:**
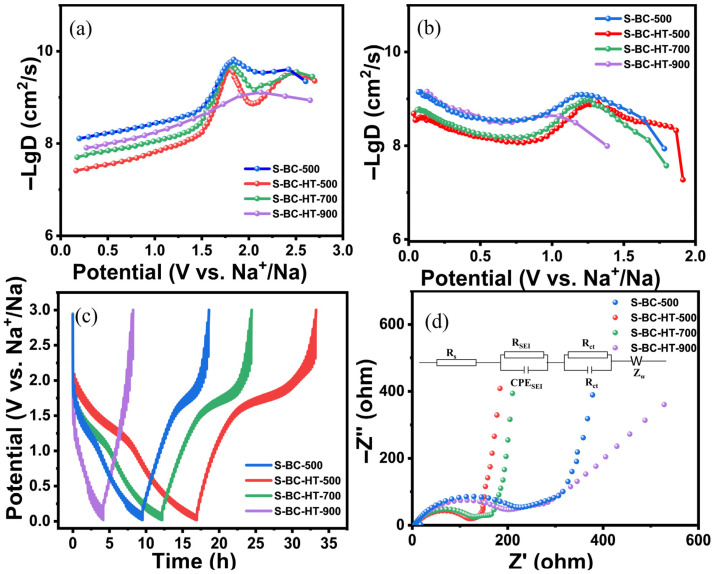
(**a**,**b**) D_Na+_ values via GITT measurements for the sedation and dissociation processes; (**c**) time–voltage curve by GITT measurements at 0.1A g^−1^; (**d**) typical Nyquist curves of S-BC-HT-500, 700, 900, and S-BC-500, respectively.

The diffusion coefficients of sodium ions were determined using the constant galvanostatic intermittent titration technique (GITT) (Figure 6c) [43,44]. The diffusion coefficient *D* is calculated as shown.

(1) $$D=\frac{4}{\pi }{\left(\frac{m_{B}V_{m}}{M_{B}S}\right)}^{2}{\left(\frac{∆E_{s}}{∆E_{t}}\right)}^{2}$$

where ∆*E_s_* is the steady-state voltage change, and ∆*E*_t_ is the total change in the battery voltage during the constant current pulse.

As shown in Figure 6a,b, the value of the diffusion coefficient of S-BC-500 is significantly lower than that of S-BC-HT-500, S-BC-HT-700, and S-BC-HT-900, which is mainly because hydrothermal dramatically changes their spatial structure and their rich specific surface area and porosity facilitate the transport of sodium ions. From the curves (Figure 6a), it can be seen that their diffusion coefficient gradually decreases with the increase in voltage, while between 1.5 and 2.2 V, their diffusion coefficient first decreases and then increases subsequently, except S-BC-HT-900, which is related to the adsorption of sulfur, and it corresponds to the Faraday reaction of sodium–sulfur, which influences the efficiency of the transport of sodium ions. As for S-BC-HT-900, there is little sulfur left as the temperature reaches 900 °C, so it hardly changes.

The excellent rate and stability properties of S-BC-HT-500 are attributed to the following three factors. Firstly, sulfur doping improves its conductivity and wettability, and the Faraday reaction of sodium and sulfur provides a capacity contribution. Secondly, the hydrothermal-treatment-induced hierarchical pore structure and large surface area offer abundant reaction sites. On one hand, they reduce the spatial resistance and enhance ion transport rates, and on the other hand, they ensure excellent contact with sulfur, thereby increasing sulfur doping in the carbon material. Finally, the doping of sulfur atoms expands the layer spacing of the carbon material, which facilitates sodium ion intercalation and extraction, thereby enhancing the storage capacity.

Using EIS to probe its charge-transfer kinetics, from Figure 6d, the impedance value consists of two main parts: the semicircle at high frequencies and the slash at low frequencies. The resistance value of the electrode material depends mainly on the diameter of the semicircle; the larger the diameter, the higher the resistance. A simplified circuit diagram of the inside of a sodium-ion battery can be seen in the inset of Figure 6d; *R_SEI_* is the SEI film resistance and the charge transfer impedance is R_ct_. Through measurements, the SEI resistance and the charge transfer resistance values of S-BC-HT-500, S-BC-HT-700, S-BC-HT-900, S-BC-500, and BC-500 are summarized in Table 1. The charge transfer resistance and the SEI resistance of S-BC-HT-500 are significantly lower than those of S-BC-500 and BC-500, which increases sequentially with an increasing heating temperature. This improvement results from the combined effects of sulfur doping and hydrothermal treatment. Hydrothermal treatment provides porosity and a specific surface area, while sulfur doping expands the layer spacing and improves the electrical conductivity [45].

Sodium ion storage in carbon materials generally follows two primary mechanisms [46]. One mechanism is the intercalation process, which is diffusion-controlled. The other one is the pseudocapacitive effect, governed by surface-limited kinetics. To elucidate the dominant sodium storage mechanism in S-BC-HT-500, CV measurements were performed at varying sweep rates from 0.1 to 5 mV s^−1^. The *b*-value is an important parameter to measure diffusion control and capacitance control, which can be calculated using the following formulas.

(2) $$i=a\nu^{b}$$

In which i represents the current, *a* and *b* are the variant, and ν is the scan rate. When the value of *b* is nearly 0.5, it shows the capacity control. In contrast, it shows the diffusive control while the value is nearly 1.

**Figure 7:**
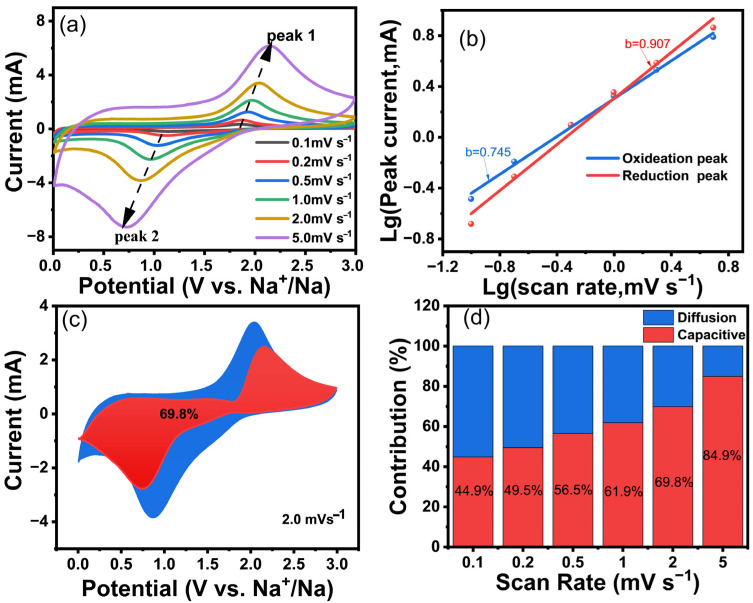
Mechanistic analysis of Na^+^-storage behavior of S-BC-HT-500 sample. (**a**) CV at different scan rates (from 0.1 to 5 mV s^−1^); (**b**) peak current corresponding to the oxidation peak; (**c**) capacitive contribution to charge storage at a scan rate of 2 mV s^−1^; (**d**) capacitive contribution to charge storage at different scan rates (from 0.1 to 5 mV s^−1^).

Meanwhile, the capacitive contribution of the electrode material can be calculated using the following equation.

(3) $$i\left(V\right)=k_{1}\nu +k_{2}\nu^{1/2}$$

where $k_{1}\nu$ represents the capacitance-controlled part and $k_{2}\nu^{1/2}$ represents the diffusion-controlled part. As shown in Figure 7a–d, the pseudocapacitance contribution of S-BC-HT-500 increases from 44.9% to 84.9% when the scan rates grow from 0.1 to 5 mV s^−1^, indicating that its storage behavior of sodium ions is mainly controlled by the pseudocapacitance. The calculated b-values of the reduced and oxidized states of S-BC-HT-500 are 0.903 and 0.745, which also explains that the storage behavior is mainly from the pseudocapacitance effect. At a scan rate of mV s^−1^, the pseudocapacitance contribution reaches 69.8%, highlighting its important role in sodium storage. In contrast, the material at a higher synthesis temperature (900 °C) exhibits a lower pseudocapacitance ratio, which also suggests that the doping of sulfur atoms enhances its pseudocapacitance contribution (Figure S3e). Overall, the sulfur-doped carbon materials exhibit enhanced pseudocapacitance properties while maintaining excellent cycling stability and versatility.

Moreover, as shown in Table 2, the shredded-coconut-derived carbon exhibits significantly higher rate capability and cycling stability compared to other recently reported biomass-derived carbon materials, demonstrating that the combination of hydrothermal treatment and sulfur doping effectively enhances its electrochemical performance [47].

## 4. Conclusions

In conclusion, we have used a facile method to convert coconut filaments into high-performance anode materials for sodium-ion batteries. The effects of pretreatment methods and heating temperatures were compared for the materials, and the effects of the pore size, layer spacing, and sulfur doping on the electrochemical properties of the materials were analyzed. The best electrochemical performance of the S-BC-HT-500 sample was found to provide a reversible capacity of 308 mAh g^−1^ at a current density of 5 A g^−1^. After 1000 cycles, it still shows 90.3% retention. Meanwhile, it showcases the capacity of 216 mAh g^−1^ at 10 A g^−1^. Its excellent cycling stability and rate performance are mainly attributed to its rich pore structure, as well as hierarchical pore structure, sulfur-doped, and large layer spacing. These characteristics facilitate diffusion, as well as the storage of sodium ions, and promote the pseudocapacitive behavior. The results demonstrate the potential of biomass materials in sodium-ion batteries and the possibility of sulfur-doped hard carbon as a sodium-ion anode material.

## Figures and Tables

**Table 1:** The resistance values of different samples.

Anode Materials	S-BC-HT-500	S-BC-HT-700	S-BC-HT-900	S-BC-500	BC-500
*R_SEI_* (Ω)	124	148	238	250	276
*R_ct_* (Ω)	86	102	191	240	279

**Table 2:** Comparison of electrochemical properties of different biomass materials.

Biomass Species	Current Density (mA g^−1^)	Cycling Number	Specific Capacity (mAh g^−1^)	The ICE (%)	Retaining Capacity (%)	References
basswood	200	500	242.3	59	94	[10]
waste coffee grounds	50	100	220.36	65	97	[11]
Switchgrass	100	100	308.4	85	98	[12]
Lotus Seedpod	50	200	328.8	50	90	[13]
Prunus armeniaca seeds	100	100	210.2	NA	NA	[14]
soap-nut seeds	100	100	154	50	69	[15]
jackfruit seed	20	100	221	66	98	[16]
chitosan	2000	500	146	50	83	[17]
kapok	400	500	185.7	75	53	[19]
sugarcane bagasse	500	1000	225.7	43	NA	[23]
cow manure	100	1000	372.2	55	70	[24]
Spring onion	5000	2000	211	58	94	[29]
Durian	5000	4500	100.02	56	NA	[30]
shredded coconut	5000	1000	308	53	90	This work

## Data Availability

The original contributions presented in this study are included in the article/Supplementary Materials. Further inquiries can be directed to the corresponding author(s).

## Supplementary Materials

The following supporting information can be downloaded at: https://www.mdpi.com/article/10.3390/nano15100734/s1, Figure S1: (a–c) the adsorption-desorption curves and pore size distributions of S-BC-HT-700, S-BC-HT-900 and BC-500; Figure S2: (a) the rate curve of BC-500 (b) the long cycle curve of BC-500 (c) CV curves under different scan rates of BC-500; Figure S3: (a–c) CV at different scan rates (from 0.1–10 mV/S) of S-BC-HT-700, S-BC-HT-900, S-BC-500. (d–f) Capacitive contribution to charge storage at different scan rates (from 0.1–5 mV/S); Figure S4: Histogram of lattice space generated from HRTEM of S-BC-HT-500; Figure S5: (a–d). The long cycle curve of S-BC-HT-500, 700, 900, S-BC-500; Figure S6: The EIS of the BC-500; Table S1: Element contents of S-BC-HT-500, 700 and 90.
