# Supplementary material for: Shredded-Coconut-Derived Sulfur-Doped Hard Carbon via Hydrothermal Processing for High-Performance Sodium Ion Anodes
Source: Nanomaterials (Basel). 2025 May 14;15(10):734. doi: 10.3390/nano15100734 (PMC12114512; doi:10.3390/nano15100734)
Supplement: Supplementary file 1 [file nanomaterials-15-00734-s001.zip › nanomaterials-3564500-supplementary.pdf]

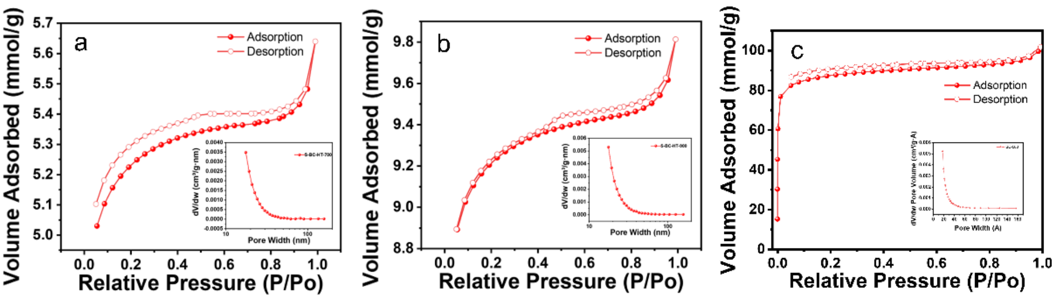

Fig. S1. a, b, c the adsorption-desorption curves and pore size distributions of S-BC-HT-700, S-BC-HT-900 and BC-500

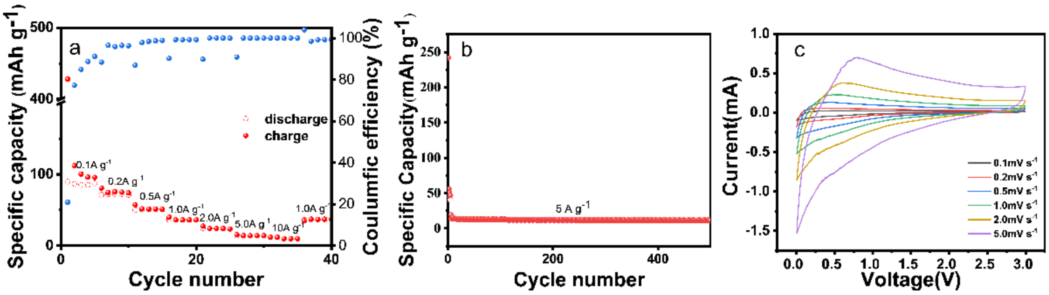

Fig S2. (a) the rate curve of BC-500 (b) the long cycle curve of BC-500 (c) CV curves under different scan rates of BC-500

sample	N(%)	C(%)	H(%)	S(%)
S-BC-HT-500	0.18	54.66	1.32	31.32
S-BC-HT-700	0.14	67.34	1.14	24.48
S-BC-HT-900	0.28	80.34	1.16	6.39

Table S1. Element contents of S-BC-HT-500, 700 and 90

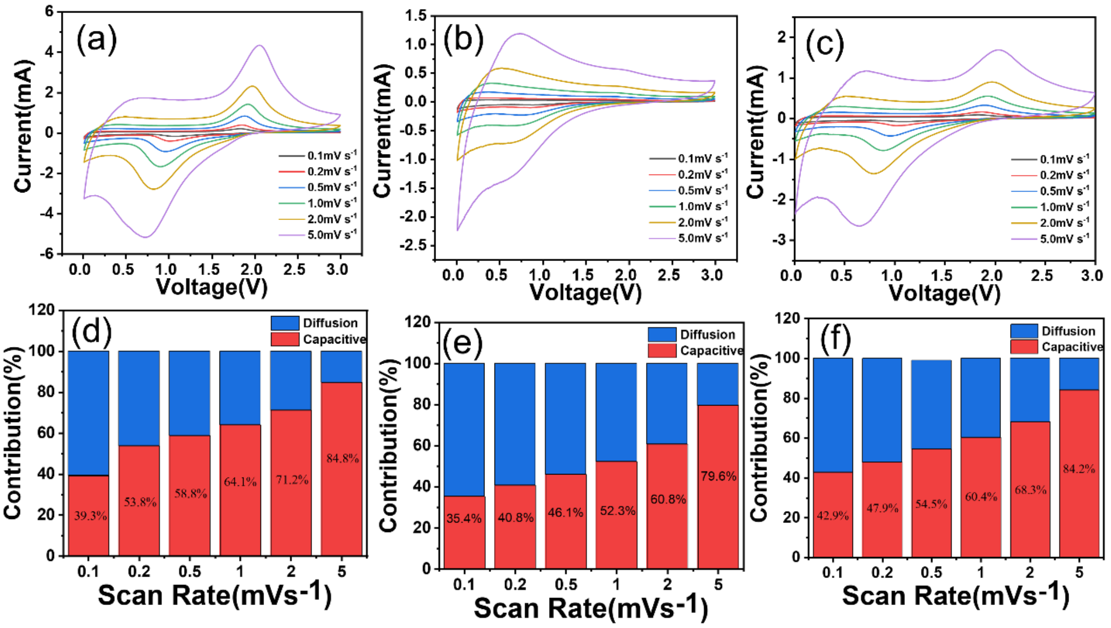

Fig. S3. (a-c) CV at different scan rates (from 0.1-10mV/S) of S-BC-HT-700, S-BC-HT-900, S-BC -500.(d-f) Capacitive contribution to charge storage at different scan rates (from 0.1-5mV/S).

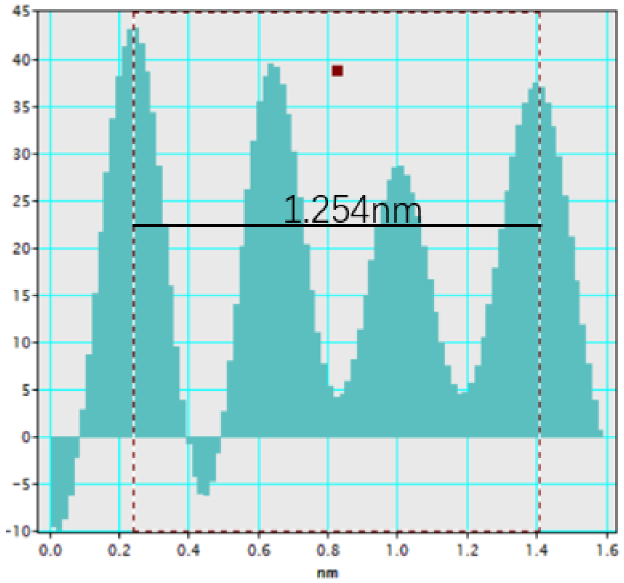

D=1.254/3=0.418nm

Fig S4. Histogram of lattice space generated from HRTEM of S-BC-HT-500

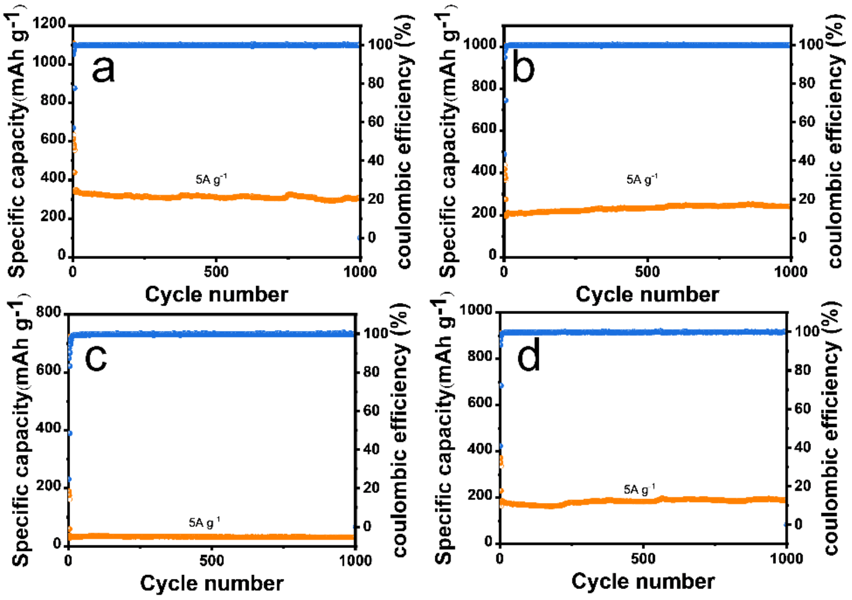

Fig S5 (a-d). the long cycle curve of S-BC-HT-500, 700, 900, S-BC-500

=

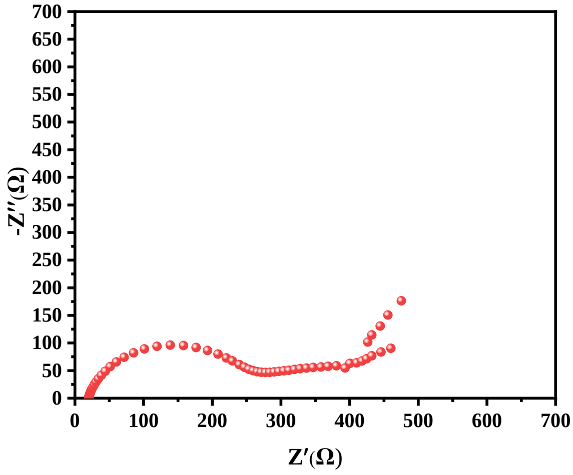

Fig. S6 the EIS of the BC-500
